# Residential stock data and dataset on energy efficiency characteristics of residential building fabrics in Ireland

**DOI:** 10.1016/j.dib.2020.105247

**Published:** 2020-02-22

**Authors:** Tomás Mac Uidhir, Fionn Rogan, Matthew Collins, John Curtis, Brian Ó Gallachóir

**Affiliations:** a Energy Policy and Modelling Group, MaREI Centre, Environmental Research Institute, University College Cork, Lee Road, Cork, Ireland; b Economic and Social Research Institute, Sir John Rogerson's Quay, Dublin, Ireland; c School of Engineering, University College Cork, Co. Cork, Ireland

**Keywords:** Residential energy efficiency database, Building energy rating, Energy performance certificates, Dwelling energy assessment procedure

## Abstract

These data support the research article “Improving energy savings from a residential retrofit policy: a new model to inform better retrofit decisions” – (Mac Uidhir et al., 2019) [1]. This article presents 3 data sources which are utilised in conjunction with a detailed energy system model of the residential sector to explore policy pathways for residential retrofitting. Data is collected from the Central Statistics Office (CSO) and the Sustainable Energy Authority of Ireland (SEAI). The first SEAI dataset is compiled for Ireland in compliance with the *EU Energy Performance of Buildings Directive* (EPBD) [2]. Data is collected using the Dwelling Energy Assessment Procedure (DEAP) [3]. DEAP is used to produce energy performance certificates known as Building Energy Ratings (BER). A BER indicates a buildings energy performance across a 15-point energy efficiency scale, rated alphabetically from A1 to G, in units of kWh/m^2^ year. A BER is required for new buildings and the rent or sale of existing dwellings – therefore the database has consistently grown in size since its inception in 2006. The BER database contains 735,906 records of individual dwellings. The database includes detailed building fabric information across a range of different building types, year of construction, Main/Secondary space/water heating fuels, heating system efficiency, ventilation method and structure type (Insulated concrete form, Masonry, Timber or Steel Frame). The second SEAI dataset (PWBER) contains aggregated pre and post BER information for a sample of 112,007 dwellings retrofitted during the period 2010–2015; this database contains mean energy efficiency improvement (kWh/m^2^ year) for a range of retrofit combinations as they apply to nine distinct building archetypes. The third CSO dataset is compiled from census data, representing the frequency of building types by year of construction.

**Table:** Specifications Table

Subject	Engineering (General)
Specific subject area	Residential dwelling energy performance characteristics and stock for Ireland.
Type of data	Microsoft SQL Database, Excel Spreadsheet with supplementary tables
How data were acquired	• BER database information was acquired from the Sustainable Energy Authority of Ireland (SEAI). Provided as unfiltered database of all BER records. Microsoft SQL used to process/query this database. • PWBER data acquired from SEAI. • National building census data gathered from Central statistics office (CSO).
Data format	Filtered model input data, SQL format Raw Model Input data
Parameters for data collection	All data on construction characteristics impacting energy performance of residential dwellings in Ireland.
Description of data collection	Data made available by the Sustainable Energy Authority of Ireland. Stored in SQL database and filtered using data collection parameters specified in section 2.1.4. CSO National Stock acquired from the Central Statistics Office (CSO)
Data source location	CSO/PWBER data related to Ireland, BER data provided provide at postal code level for Dublin and City/County level for all other counties.
Data accessibility	*Data is provided with this article in the following formats:* • BER Database is provided with the article in the form of SQL database attachment. • PWBER Data provided as supplementary Excel file • CSO data is provided within this article
Related research article	T. Mac Uidhir, F. Rogan, M. Collins, J. Curtis, B. Ó Gallachóir. Improving energy savings from a residential retrofit policy: a new model to inform better retrofit decisions. Energy and Buildings. https://doi.org/10.1016/j.enbuild.2019.109656 [1]

**Table:** 

**Value of the Data** • This data provides transparency to model input parameters used in the evaluation of energy efficiency measures for residential dwellings in Ireland. The data provides a detailed source of building fabric information in a queryable format. • Energy analysts can benefit from the detailed building fabric information, serving to aid in replication of residential energy efficiency analyses. Policymakers can also benefit from detailed analyses underpinning evidence-based policy support. • This data can be used to gain insights into the link between energy performance of specific building fabrics and the associated net improvement to building energy efficiency.

## 1 Data

The supplementary SQL database attachment provided with this article contains detailed building fabric performance characteristics for 735,906 dwelling records. Informational data is provided for each record in the form of a description of the dwelling type (Apartment, Basement Dwelling, Detached house, End of terrace house, Ground-floor apartment, House, Maisonette, Mid-floor apartment, Mid-terrace house, Semi-detached house, Top-floor apartment), year of construction, dwelling location (postal code for Dublin and City/County description for all other counties), date/purpose of the BER assessment (Grant Support, New Dwelling, Private Letting, Sale, Social Housing Letting, Unknown, Other). Building fabric data is provided in the form of U-Values (W/m^2^ K) and surface area (m^2^) for each dwelling's walls, roof, floors, windows and doors. The number of building stories, ground floor area (m^2^), heating system efficiency and the main/secondary space/water heating fuels are also provided for each record. This data is gathered for Ireland in compliance with the EU EPBD [2] using DEAP [3].

The datasets within this article provide CSO census [4] and BER data on the number of dwellings by type, year of construction and BER grade category (Table 2). This data is presented in Table 1 and Table 2.

**Table 1:** CSO Census data, number dwellings by type and year of construction.

House Type	<1919	1919 to 1945	1946 to 1960	1961 to 1970	1971 to 1980	1981 to 1990	1991 to 2000	2001 to 2005	>2006	Not stated
Detached house	74125	46847	42427	42221	97698	86491	111455	175223	18050	20596
Semi-detached house	15478	25149	39121	43364	71056	49522	80437	115869	5900	26052
Terraced house	36956	31594	38410	24370	36397	23557	19161	51682	3127	19315
Flat or apartment in a purpose-built block	3159	2434	3415	4039	5873	9310	27108	84521	5717	26520
Apartment in converted house/commercial building	9575	2653	1745	1186	1176	1039	1530	2247	365	7267
Bed-sit	972	306	229	195	152	136	142	152	42	940
Not stated	935	685	760	666	1121	989	978	2069	235	13432

**Table 2:** BER data: Number of dwelling archetypes by type, year of construction and BER group.

Building Archetype	<1919	1919 to 1945	1946 to 1960	1961 to 1970	1971 to 1980	1981 to 1990	1991 to 2000	2000 to 2005	>2006
Apartment AB	132	296	185	422	249	180	964	6407	22054
Apartment CD	1769	1065	1044	1450	2442	4091	17611	31561	19926
Apartment EFG	7216	2371	1615	1500	1963	3187	8587	6397	2212
Terrace AB	644	893	1198	904	1757	1759	2796	4909	31761
Terrace CD	6401	9590	12820	12818	30992	30395	45837	50996	22915
Terrace EFG	13929	13619	15040	8137	13195	7499	7831	3727	1088
Detached AB	393	355	404	400	944	1195	3173	4929	15399
Detached CD	3777	3118	3598	4883	16510	18647	31420	26730	9761
Detached EFG	11400	8329	6755	5432	9209	4881	3163	1149	573
All Types	45661	39636	42659	35946	77261	71834	121382	136805	125689

Data specifying the total number of dwelling types, by year of construction, is presented in Table 1. This data was collected as part of the national census completed in 2016. The energy performance of building types is not included in this data.

Data specifying the total number of building archetypes, by year of construction and energy performance grouping is presented in Table 2. This dataset is collected as part of Building Energy Rating (BER) programme operated by SEAI. A BER is compulsory for all new dwellings, dwellings being sold/rented, dwellings in receipt of an SEAI energy efficiency grant.

The BER database, included as supplementary material, represents a range of 140 individual building characteristics as they apply to 735,906 dwellings. The average U-Value (W/m^2^K) for walls, roof and windows, for each of the nine dwelling archetypes and year of construction bracket, is shown in Table 3, Table 4, Table 5 respectively. A complete list of building characteristics is included and further described in Table 7.

**Table 3:** BER data: average U-value (W/m^2^K) for **external walls** by dwelling type, BER category and year of construction grouping.

Building Archetype	<1919	1919 to 1945	1946 to 1960	1961 to 1970	1971 to 1980	1981 to 1990	1991 to 2000	2000 to 2005	>2006
Apartment AB	0.50	0.38	0.37	0.65	0.48	0.46	0.52	0.47	0.34
Apartment CD	1.06	0.98	0.99	0.96	0.78	0.55	0.57	0.54	0.43
Apartment EFG	1.59	1.59	1.64	1.52	1.20	0.64	0.64	0.58	0.54
Terrace AB	0.38	0.34	0.33	0.34	0.38	0.38	0.38	0.38	0.24
Terrace CD	1.12	0.92	0.94	0.85	0.72	0.48	0.47	0.45	0.35
Terrace EFG	1.77	1.73	1.76	1.62	1.32	0.54	0.44	0.39	0.37
Detached AB	0.36	0.31	0.30	0.30	0.33	0.35	0.38	0.37	0.25
Detached CD	0.95	0.81	0.70	0.67	0.60	0.46	0.44	0.44	0.35
Detached EFG	1.70	1.65	1.53	1.36	1.13	0.58	0.49	0.45	0.44

**Table 4:** Average U-value (W/m^2^K) for **roof** by dwelling type, BER category and year of construction grouping.

Building Archetype	<1919	1919 to 1945	1946 to 1960	1961 to 1970	1971 to 1980	1981 to 1990	1991 to 2000	2000 to 2005	>2006
Apartment AB	0.12	0.07	0.08	0.19	0.13	0.09	0.10	0.10	0.08
Apartment CD	0.29	0.24	0.29	0.46	0.22	0.15	0.15	0.14	0.12
Apartment EFG	0.94	0.98	0.92	0.84	0.50	0.23	0.21	0.18	0.18
Terrace AB	0.22	0.21	0.22	0.20	0.19	0.18	0.19	0.21	0.15
Terrace CD	0.57	0.45	0.42	0.39	0.28	0.25	0.27	0.25	0.21
Terrace EFG	1.46	1.15	1.00	0.84	0.58	0.31	0.26	0.22	0.48
Detached AB	0.21	0.20	0.20	0.21	0.20	0.22	0.24	0.23	0.17
Detached CD	0.47	0.41	0.36	0.36	0.28	0.25	0.27	0.27	0.25
Detached EFG	1.30	1.15	0.95	0.77	0.57	0.36	0.34	0.41	0.74

**Table 5:** Average U-value (W/m^2^K) for **windows** by dwelling type, BER category and year of construction grouping.

Building Archetype	<1919	1919 to 1945	1946 to 1960	1961 to 1970	1971 to 1980	1981 to 1990	1991 to 2000	2000 to 2005	>2006
Apartment AB	2.60	2.35	2.22	2.31	2.05	2.62	2.63	2.40	1.85
Apartment CD	3.08	3.01	2.85	2.78	2.82	2.87	2.89	2.60	2.25
Apartment EFG	3.53	3.44	3.39	3.39	3.34	3.17	2.96	2.68	2.32
Terrace AB	2.08	1.97	1.91	2.25	2.23	2.31	2.33	2.38	1.62
Terrace CD	2.93	2.77	2.82	2.87	2.88	2.93	2.85	2.63	2.25
Terrace EFG	3.70	3.58	3.59	3.37	3.21	3.18	2.97	2.77	2.31
Detached AB	1.97	1.83	1.79	1.86	2.06	2.35	2.58	2.43	1.70
Detached CD	2.81	2.74	2.76	2.82	2.85	2.88	2.83	2.61	2.26
Detached EFG	3.57	3.47	3.45	3.36	3.29	3.32	2.99	2.75	2.33

**Fig. 1:**
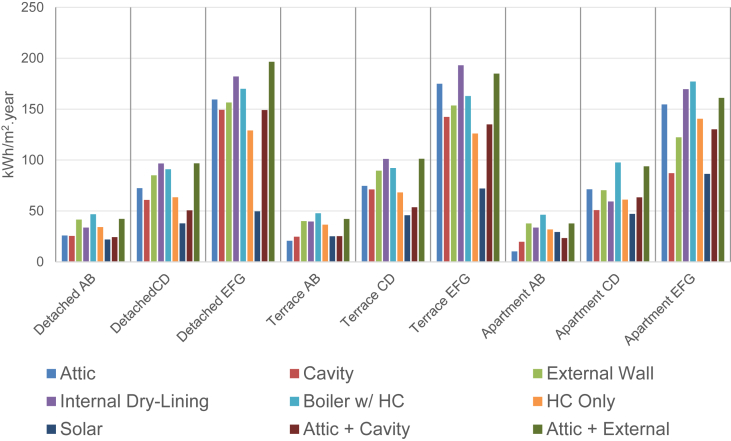
PWBER Data: Average annual energy efficiency improvement by retrofit combination and dwelling archetype.

The PWBER dataset included as supplementary material represents the average energy efficiency improvement (kWh/m^2^ year), for a range of 50 retrofit combinations, as they apply to nine distinct building archetypes. These archetypes include energy performance groupings (AB, CD, EFG) applied to apartment, detached and terraced dwellings. Fig. 1 illustrates the average annual energy savings (kWh/m2 year) associated with nine distinct retrofit combinations from the PWBER dataset.

## 2 Experimental design, materials, and methods

This section outlines the steps required to acquire, process and analyse the data referenced in this article.

### 2.1 Census data on housing in Ireland

The CSO provide direct access to 2016 census results for building type by year of construction through an online portal [4]. CSO survey definitions for building type differ from other sources and are therefore aggregated into three building types (Detached, Terraced, Apartment), as shown below in Table 6.

**Table 6:** CSO dwelling type definitions - census 2016.

Dwelling Type (CSO)	Dwelling Type Aggregated
Detached House	Detached
Semi-Detached House	Terraced
Terraced House	
Flat or Apartment in purpose-built block	Apartment
Flat or Apartment in converted house or commercial building	
Bed-Sit	N/A
Not Stated	N/A

### 2.2 BER database

This process describes the acquisition and filtering procedures to produce the included BER input database. Table 2, Table 3, Table 4, Table 5 are derived directly from the filtered BER database.

2.1.1 The Sustainable Energy Authority of Ireland host a public national depository of all BER records, available for download in excel format [5]. This format is not suitable for analysis and required further processing to produce queryable database in SQL format.
2.1.2 This Raw Data is imported into a blank Microsoft SQL database table using SQL Server Integration Services (SSIS). SSIS is used for complex data transformation and managing/filtering data [6]. This process allows all 735,906 records to be queried individually. A series of scripts are then utilised to manage and filter the database, adding unique record ID's for each record in the database and removing unwanted outliers. Each script is provided with this article and its function described here.
2.1.3 Using SQL Server Management studio (SSMS) [7]. A unique ID is associated with each record in the BER database. Executing SQL Script 1 creates a new database table which includes a record ID column and inserts all other records accordingly. This record ID is used to track deleted records upon removal of outliers. The ID is helpful with respect to error handling and understanding the reason an individual record might be removed.
2.1.4 Outliers are removed from the database, removing any records which satisfy the following criteria; Results are provisional, Main floor area = 0 m^2^, Ground floor area ≤30 m^2^, Ground floor area >1000 m^2^, Apartments/Terraced Dwellings with floor area >500 m^2^, Heating System Efficiency <19%, Heating System Adjustment Factor <0.7, Main Water Heating system efficiency >450%, Main Water Heating system efficiency <19%, Water Heating Efficiency Adjustment Factor <0.7, Living Area Percentage >90, Living Area Percentage <5, Supplementary Heat Fraction ∉ {0,0.1,0.15,0.2}, Declared Loss Factor >20, Thermal Bridging Factor <0, Thermal Bridging Factor >0.15, Dwelling Type Description ∈ {House, Basement Dwelling, Maisonette} – resulting in removal of 46,661 records.
Executing SQL Script 2 removes record outliers from the database, tracking the total number of records removed from the database for each criterion stated.
2.1.5 Executing SQL Script 3 creates the final table and inserts all relevant values from the processed database. This table forms the input data for use within the energy system model defined as the SQL Archetype Dwelling Energy Model (ArDEM-SQL) [1]. Table 7 shows the complete list of input variables in this final table. The complete database backup is included as supplementary SQL backup (Backup.bak).

**Table 7:** Data input variables name and description.

SQL input variable name	SQL input variable description
Record ID	Unique BER record identifier
CountyName	BER record geographical location (county)
DwellingTypeDescr	Description of dwelling type e.g. Detached, Apartment
Year_of_Construction	Building year of construction
TypeofRating	Nature of BER record, Final, Existing or Provisional
EnergyRating	Letter grade for energy performance e.g. A1, A2, A3, B1, B2, B3, C1, C2, C3, D1, D2, E1, E2, F, G
BerRating	Numerical energy performance rating (kWh/m^2^.year)
GroundFloorArea(sq m)	Ground Floor Area (m^2^)
UValueWall	Wall U-Value (W/m^2^K)
UValueRoof	Roof U-Value (W/m^2^K)
UValueFloor	Floor U-Value (W/m^2^K)
UValueWindow	Window U-Value (W/m^2^K)
UvalueDoor	Door U-Value (W/m^2^K)
WallArea	Wall Area (m^2^)
RoofArea	Roof Area (m^2^)
FloorArea	Floor Area (m^2^)
WindowArea	Window Area (m^2^)
DoorArea	Door Area (m^2^)
NoStoreys	Number Storeys per dwelling
CO2Rating	BER CO2 intensity rating (kgCO2/m^2.^yr)
MainSpaceHeatingFuel	Predominant fuel used for Main Space Heating
MainWaterHeatingFuel	Predominant fuel used for Main Water Heating
HSMainSystemEfficiency	Main Heating System Efficiency (%)
HSEffAdjFactor	Heating system energy efficiency adjustment factor
HSSupplHeatFraction	Supplementary Heating system fraction of heating requirement
HSSupplSystemEff	Supplementary heating system efficiency (%)
WHMainSystemEff	Main Water heating System Efficiency (%)
WHEffAdjFactor	Water Heating Efficiency Adjustment Factor
SupplSHFuel	Supplementary Space Heating fuel
SupplWHFuel	Supplementary Water Heating Fuel
NoOfChimneys	Number of Chimney stacks in dwelling
NoOfOpenFlues	Number of Open Flues in dwelling
NoOfFansAndVents	Number of fans and vents in dwelling
NoOfFluelessGasFires	Number of Gas Fires not including Flues
DraftLobby	Is a draft lobby present on entrance (yes/no)
VentilationMethod	Dwelling ventilation method e.g. Natural Ventilation
StructureType	Masonry, Timber or Steel frame
SuspendedWoodenFloor	Is there a suspended wooden floor in dwelling (yes/no)
PercentageDraughtStripped	Percentage of floor draught stripped (%)
NoOfSidesSheltered	Number of sheltered walls
PermeabilityTest	Was a permeability test performed (yes/no)
PermeabilityTestResult	Permeability test result (m^3^/hour)
TempAdjustment	Applied space heating temperature adjustment - dependent on space heating control category (°C)
HeatSystemControlCat	Heating system control category ID
HeatSystemResponseCat	Heating system response category ID
NoCentralHeatingPumps	Number of central heating pumps
UndergroundHeating	Does dwelling utilise underfloor heating (yes/no)
GroundFloorUValue	Ground floor U-Value (W/m^2^K)
DistributionLosses	Hot water heating distribution losses - dependent on hot water storage insulation (kWh/year)
StorageLosses	Hot water storage losses
SolarHotWaterHeating	Is solar water heating used in dwelling (yes/no)
ElecImmersionInSummer	Supplementary electric immersion used in summer months (yes/no)
CombiBoiler	Is Combi boiler used in dwelling (yes/no)
WaterStorageVolume	Hot water storage volume (L)
InsulationType	Hot water storage insulation type e.g. Loose Jacket
InsulationThickness	Hot water storage insulation thickness (mm)
PrimaryCircuitLoss	Hot water primary circuit losses (kWh/year)
GroundFloorArea	Total ground floor area (m^2^)
GroundFloorHeight	Total ground floor height (m)
FirstFloorArea	Total first floor area (m^2^)
FirstFloorHeight	Total first floor height (m)
SecondFloorArea	Total second floor area (m^2^)
SecondFloorHeight	Total second floor height (m)
ThirdFloorArea	Total third floor area (m^2^)
ThirdFloorHeight	Total third floor height (m)
ThermalBridgingFactor	Transmission heat loss due to thermal bridging (W/m^2^.K)
ThermalMassCategory	Index of heat capacity required, rated ow, medium-low, medium, medium-high or high
PredominantRoofTypeArea	Total area of main roof (m^2^)
PredominantRoofType	Total main roof construction type
LowEnergyLightingPercent	Percentage of energy efficiency lighting installed (%)
LivingAreaPercent	Percentage of building used for living space (%)
RoomInRoofArea	Is attic converted to living space (yes/no)
MainFloorArea	Total dwelling floor area (m^2^)
PurposeOfRating	Reason for BER assessment e.g. Sale, Retrofitting
DateOfAssessment	Date of BER assessment
